# Estimating the Susceptibility of Four Wheat Cultivars to the Saw-Toothed Grain Beetle *Oryzaephilus surinamensis* (L.) (Coleoptera: Silvanidae)

**DOI:** 10.1155/2022/8570732

**Published:** 2022-08-21

**Authors:** Fatehia Nasser Gharsan

**Affiliations:** Department of Biology, Faculty of Science, Albaha University, 2036-65654, Saudi Arabia

## Abstract

The quality and quantity of wheat are severely affected by insect infestation during storage. Excessive use of chemical pesticides has resulted in several environmental issues, in addition to the emergence of pesticide-resistant strains. Extensive research has been conducted to determine sustainable alternatives, such as the selection and cultivation of insect-resistant crop varieties. The aim of this study was to determine the degree of susceptibility of four wheat cultivars, namely, Albelad, Aldwasair, Australia, and Najran (NJ), to Oryzaephilus surinamensis (L.) using four parameters: F1 progeny, length of developmental period, Dobie's index of susceptibility (DI), and percentage weight loss (PWL). Albelad was highly susceptible (DI =17.44) to O. surinamensis, followed by Najran (DI =8.42) and Australia (DI =6.18) (susceptible and moderately susceptible, respectively), whereas Aldwasair was moderately resistant (DI =4.67). Furthermore, there were significant positive correlations between DI and the mean number of F1 progeny (*R*^2^ = 0.98) and between DI and grain weight loss (*R*^2^ = 0.95). However, a significantly negative correlation (*R*^2^ = −0.86) was observed between DI and beetle developmental period. These findings will aid the development of breeding programs to maintain the quality and quantity of wheat grains during storage.

## 1. Introduction

Wheat is one of the most important crops worldwide, as it is one of the vital sources of nutrition and is used in several food industries. Generally, it is stored until exported, sold, or used for consumption. However, after harvesting, wheat is exposed to several agents during storage that may alter its quality and quantity and render it unsuitable for human consumption by the time it reaches the consumer [1–3]. Among the agents that affect stored grains are insect pests that cause an estimated global yield loss of 5–10%; yield losses of 0.05–3% and 45–50% have been reported in developed and developing countries, respectively [4, 5]. Although the contribution of the Albaha region to the country's wheat production is low, there are still people who depend on the wheat that they grow on their farms. Albelad wheat is the most common cultivar in the Albaha region, and therefore, its susceptibility to being infested with one of the stored products insects was compared with three other cultivars. The saw-toothed grain beetle, *Oryzaephilus surinamensis* (L.) (Coleoptera: Silvanidae), is an insect pest that infests a wide range of stored crops, including wheat and crop products in approximately all storage facilities [6]. Therefore, it is necessary to develop strategies to eradicate this warehouse pest.

Chemical pesticide application is one of the most common methods used for pest control. However, despite their short-term efficacy, these pesticides cause several environmental problems. Furthermore, they may be toxic to non-target organisms and result in the emergence of pesticide-resistant strains [7]. Thus, extensive research has been conducted to develop safer alternatives to reduce environmental pollution and support sustainable agriculture. Among these sustainable agriculture strategies, identifying and cultivating pest-resistant crop varieties are paramount.

Different varieties of stored crop products differ in their susceptibility to *O. surinamensis* infestation [8, 9]. Several parameters have been used to determine the susceptibility of stored crops to warehouse pests, with the susceptibility index being the most important. Different wheat varieties are susceptible to infestation by different warehouse pests at varying degrees [2, 10–12]. However, to the best of our knowledge, there have been no studies regarding wheat cultivars resistant to *O. surinamensis* in Saudi Arabia. Therefore, this study aimed to determine the susceptibility of four wheat cultivars grown in the Albaha region to *O. surinamensis* using the following four parameters: F_1_ progeny, the length of developmental period, Dobie's index of susceptibility (DI), and percentage weight loss (PWL). We also determined the correlation between DI and other parameters. The findings will provide vital information for pest control strategies in wheat and help reduce the use of chemicals to achieve one goal of sustainable agriculture that benefits the farming and scientific communities.

## 2. Materials and Methods

### 2.1. Wheat Cultivars

Commercial wheat cultivars used in the study were planted in the Albaha region (20° 0′ N, 41° 30′ E) of the Kingdom of Saudi Arabia in 2017. We used three local wheat cultivars, namely, “Albelad” from Albaha, “Aldwasair” from Wadi Al Dawasir, and “Najran” from Najran city, and an imported “Australia cultivar.”

### 2.2. The Weight of a Thousand Wheat Cultivars (gm)

Thousand grains of each cultivar were weighed. The grains were counted manually; then, the grains were weighed.

### 2.3. Protein and Total Lipid Estimation

The percentage of protein was estimated according to the method used in AACC [13], while the percentage of total fat was estimated using the method of AOAC [14].

### 2.4. Insect Rearing

*O. surinamensis* was collected from infested crop products obtained from a local market in Albaha city, Saudi Arabia, and reared under laboratory conditions [27°C, 70% ±5% RH, and a 12 : 12 h (L:D) photoperiod]. Yeast and flour (5 : 100 g) were used to feed the insects, which were reared in glass jars (1,000 mL) covered with muslin cloth and fastened with rubber bands. The life cycle of insects from laying eggs to the emergence of adults extended for 30 d under ideal conditions. One- to three-week-old adults were collected and used for subsequent experiments [15].

### 2.5. Progeny Emergence and Determination of Developmental Period

To determine the number of F_1_ progeny, 10 pairs of *O. surinamensis* adults were reared on different wheat cultivars in containers for 5 d to allow mating and oviposition. The adults were then removed, and the containers were transferred to incubators at 27°C ±1°C and 70% ±5% RH. After seven weeks, the number of F_1_ adults emerging from the grains of each wheat cultivar was estimated. The beetle developmental period was determined as the time from oviposition to the emergence of 50% of the F_1_ progeny [16].

### 2.6. Estimation of Susceptibility Index

DI was calculated for each cultivar using the following formula [16]:

(1) $$\begin{array}{c} \text{DI}=\left(\log_{e}F\right)/\text{MDP}\times 100, \end{array}$$

where *F* is the total number of emerged adults, log_*e*_ is the natural logarithm, and MDP is the median developmental period (estimated as the number of days from the middle of the oviposition period to the emergence of 50% of F_1_ adults).

DI has been used to classify cowpea cultivars into different categories [16, 17] according to the following scale: <4.1: highly resistant; 4.1–6.0: moderately resistant; 6.1–8.0: moderately susceptible; 8.1–10: susceptible; and >10: highly susceptible. The same scale was used to categorize the susceptibility of wheat cultivars in this study.

### 2.7. Percentage Weight Loss of Wheat Grains

Fifty grams of wheat grains were placed in 500-mL containers with 10 pairs of insects for 10 d for oviposition. Subsequently, pest-infested grains were removed and stored for three months.

PWL index was calculated for each cultivar using the following formula [18]:

(2) $$\begin{array}{c} \left(\text{Initial grain weight}–\text{Final grain weight}\right)/\text{Initial grain weight}\times 100. \end{array}$$

### 2.8. Statistical Analyses

All experiments were performed in triplicates, and the mean values and standard deviation were estimated using SPSS 21.0 for Windows (SPSS Inc., Chicago, IL, USA). Statistical comparisons were performed using one-way analysis of variance and Duncan's multiple range test at *p* < 0.05. Pearson's correlation coefficient analysis was used to determine the correlation between DI and other parameters.

## 3. Results

### 3.1. The Weight of a Thousand Wheat Cultivars (gm)

The results in Table 1 indicate the difference in weights between wheat cultivars per 1000 grains. Aldwasair variety recorded the highest weight, reaching 49.3 gm/1000 grains. There were significant differences between Aldwasair cultivar and the rest of the cultivars except for the Australia cultivar, where the average weight was 43.1 gm/1000 grains, then Najran cultivar (36.9gm/1000 grains), and finally, Albelad cultivar 34 gm/1000 grains.

### 3.2. Protein and Total Lipid Estimation

There are differences between wheat cultivars in the percentages of protein and lipid, and for each but there are no statistically significant differences between the cultivars where the *p* value was >0.05 (Table 2).

### 3.3. F_1_ Progeny Emergence and Developmental Period

Table 3 shows that the average number of F_1_*O. surinamensis* progeny varied among wheat cultivars. Albelad recorded the highest number of F_1_ progeny (104), whereas Aldwasair recorded the lowest (7.33). Furthermore, the developmental period (26.67 d) of insects infesting Albelad was shorter than that of insects infesting other cultivars. By contrast, the longest beetle developmental period was recorded for Aldwasair (42.67 d). For the Australia and Najran cultivars, the average number of F_1_ progeny was 8 and 22.67, respectively, and the developmental period extended to 33.67 d and 37.33 d, respectively. Statistical analyses revealed significant differences in the average number of F1 progeny among all the cultivars, except Najran and Australia (*p* < 0.05).

### 3.4. DI Estimation

DI of the wheat cultivars ranged from 4.67 in Aldwasair to 17.66 in Albelad. Thus, the Albelad cultivar was categorized as highly susceptible to *O. surinamensis* infestation, whereas Aldwasair was moderately resistant. The Najran and Australia cultivars were categorized as susceptible (8.42) and moderately susceptible (6.18), respectively. Furthermore, significant differences were observed in the mean DI values among the wheat cultivars (*p* < 0.05) (Table 4).

### 3.5. PWL Indices of Wheat Cultivars

The highest and lowest PWL indexes were observed in the Albelad (23.13%) and Australia (0.2%) cultivars, respectively. Moreover, there were significant differences between the PWL indexes of Albelad and other cultivars (*p* < 0.001). However, no significant differences were observed among the other three cultivars (Table 5).

### 3.6. Relationship between DI and Other Parameters

**Figure 1:**
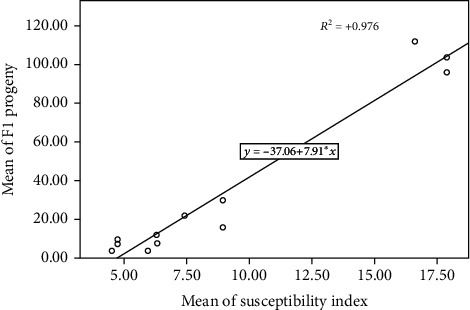
Correlation between Dobie's index of susceptibility (DI) and number of F1 progeny.

**Figure 2:**
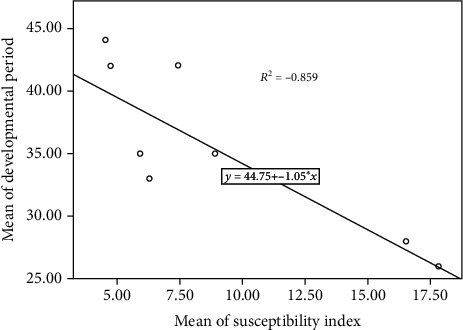
Correlation between DI and beetle developmental period.

**Figure 3:**
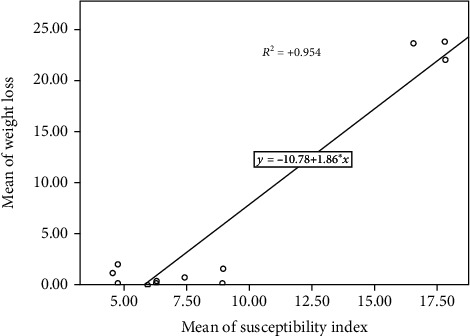
Correlation between DI and percentage weight loss.

DI was significantly positively correlated with the mean number of F1 progeny (*R*^2^ = 0.976) (Figure 1). However, a significant negative correlation was observed between DI and beetle developmental period (*R*^2^ = −0.859) (Figure 2). Nonetheless, PWL was significantly positively correlated with DI (*R*^2^ = 0.954) (Figure 3).

## 4. Discussion

The results of the current study showed that the wheat cultivars differ in their susceptibility to infection by *O. surinamensis*; among all the cultivars, Aldwasair (DI =4.67) showed the highest resistance, whereas Albelad (DI =17.44) showed the highest susceptibility to insect infestation. The level of susceptibility of varieties to insects is determined through several parameters, including the number of F_1_ progeny, insect developmental period, and grain weight loss [2, 11, 19]. Moreover, the correlation coefficient was determined between the level of susceptibility of different cultivars to *O. surinamensis* infestation and various parameters. DI was found to be significantly positively correlated to the mean number of F_1_ progeny and grain weight loss. However, a significantly negative correlation was observed between DI and beetle developmental period. These results are consistent with those reported by Rajput et al. [11], which showed a positive correlation between adult population and percent infestation, percent weight loss. Furthermore, the highest and lowest PWL indexes observed in the Albelad and Australia cultivars are consistent with those reported by Sayed et al. [20], who suggested that grain weight loss was correlated with the number of F_1_ progeny in wheat cultivars infected with *Trogoderma granarium* Everts and *R. dominica*. Moreover, Zulfikar et al. [12] associated the increase in weight loss in wheat cultivars with an increase in the number of F_1_ adults; insect numbers increased when wheat grains were stored, which resulted in grain weight loss. This may be attributed to the fact that durum wheat grains do not contain defense factors against insect infestation, unlike other plant tissues that contain defensive chemicals, such as phenols, saponins, and alkaloids. Previous studies indicated that the resistance and susceptibility of stored grains or crop products to warehouse insects are associated with several factors, such as the hardness of grains [21], genetic differences between varieties [22, 23], and chemical composition, including the composition of proteins, carbohydrates, enzymes, and starch [8]. Batta et al. [24] attributed the resistance of some wheat cultivars against *Rhizopertha dominica* (Fab.) to the lower and higher levels of proteins and carbohydrates, respectively, compared to that of sensitive cultivars. In contrast to traditional agricultural practices that rely on the excessive use of chemical pesticides, we used one of the principles of sustainable agriculture, that is, improving crop yield and profits while reducing the use of chemical pesticides [25] and replacing their use with that of pest-resistant cultivars.

Although this study identified the most *O. surinamensis*-resistant wheat cultivar (Aldwasair), all experiments were conducted under laboratory conditions. Therefore, further investigation under realistic storage conditions needs to be performed using more pest species to determine the most resistant wheat cultivar cultivated in Saudi Arabia and improve the understanding of the mechanisms underlying pest resistance in these cultivars by identifying other resistance-related factors.

## 5. Conclusions

In this study, we identified the most resistant cultivar Aldwasair that can be used in pest management programs aimed at reducing yield loss owing to *O. surinamensis* attack. Furthermore, we evaluated other less susceptible wheat cultivars, which can be stored for a longer period. Therefore, to attain the goal of sustainable agriculture, plant breeders should be encouraged to provide insect-resistant wheat cultivars, including the ones identified in this study, to farmers.

## Figures and Tables

**Table 1:** Mean weights of wheat varieties/1000 grains.

	Mean of weight ± S.E.	*p* values			
		DW	AUS	NJ	BE
Aldwasair	49.3 ± 0.18a		0.22	0.01^∗^	0.003^∗^
Australia	43.1 ± 0.2 ac			0.215	0.05^∗^
Najran	36.9 ±1.03c				0.730
Albelad	34 ± 3.9bc				

^∗^ The mean difference is significant at the 0.05 level.

**Table 2:** Protein and total lipid estimation.

Cultivars	Means of protein percent	Means of lipid percent
Albelad	0.16 ± 0.07a	0.03 ± 0.02a
Aldwasair	0.096 ± 0.04a	0.02 ± 0.01a
Australia	0.14 ± 0.04a	0.004 ± 0.002a
Najran	0.11 ± 0.06a	0.01 ± 0.001a

**Table 3:** Number of F_1_ progeny and the length of developmental period of *Oryzaephilus surinamensis* (L.) reared on four wheat cultivars upon 5 d of egg-laying by 10 female adults.

Wheat cultivars	Number of F_1_ progeny (mean ± SE)	Standard deviation	Developmental period (mean ± SE)
Albelad	104 ± 4.62a^∗^	1.15	26.67 ± 0.67c
Aldwasair	7.33 ±1.76d	1.15	42.67 ± 0.67a
Australia	8.00 ± 2.31bd	1.15	33.67 ± 0.67b
Najran	22.67 ± 4.06b	0.04	37.33 ± 2.33ab

^∗^Different letters within each column indicate significantly different means (*p* < 0.05).

**Table 4:** Dobie's index of susceptibility (DI) of four wheat cultivars.

Wheat cultivars	Standard deviation	DI (means ± SE)	Level of susceptibility
Albelad	0.73	17.44 ± 0.42a^∗^	Highly susceptible
Aldwasair	0.12	4.67 ± 0.07d	Moderately resistant
Australia	0.21	6.18 ± 0.12c	Moderately susceptible
Najran	0.86	8.42 ± 0.50b	Susceptible

^∗^Different letters within each column indicate significant difference (*p* < 0.05).

**Table 5:** Percentage weight loss (PWL) of wheat grains.

Wheat cultivars	Weight loss (g) after 7 weeks	Weight loss (%)	PWL (mean ± SE)
Albelad	11.8	23.6	23.13 ± 0.28a^∗^
	11	22	
	11.9	23.8	

Aldwasair	0.6	1.2	1.13 ± 0.52b
	0.1	0.2	
	1	2	

Australia	0	0	0.2 ± 0.11b
	0.2	0.4	
	0.1	0.2	

Najran	0.8	1.6	0.87 ± 0.41b
	0.1	0.2	
	0.4	0.8	

^∗^Different letters within each column indicate significant difference (*p* < 0.05).

## Data Availability

Data supporting the study findings are included within the article. The raw data will be provided on request.
